# Assessing the Predictive Power of Self-Care and Psychological Flow on Occupational Stress in Nurses

**DOI:** 10.12688/f1000research.152259.2

**Published:** 2025-02-28

**Authors:** Abdallah Salem Almahaireh, Baha' Suhail Shawaqfeh

**Affiliations:** 1 Counseling, The University of Jordan, Amman, Jordan; 2 Counseling, Jordanian Ministry of Education, Amman, Jordan

**Keywords:** Self-Care, Psychological Flow, Occupational Stress, Nurses

## Abstract

**Background:**

The nursing workforce is crucial in healthcare systems worldwide and contributes to community well-being. also, Nurses experience numerous psychological, social, emotional, and behavioral shocks and challenges in their work. In Jordan, 43% of doctors and nurses suffer from high levels of burnout, around 55% experience high levels of emotional exhaustion, and 33% of doctors and nurses suffer from high levels of psychological pressure and job stress. Others found that 70% of nurses experience stress. these issues can be reduced by practicing self-care and psychological flow which impact in work-related tasks which are important in effectively addressing occupational stress healthily and soundly. This study aims to determine assessing the predictive power of self-care and psychological flow in occupational stress among nurses

**Methods:**

This study follows the quantitative technique. sample consisting of 476 nurses in Amman Jordan was chosen. Three scales were adapted to meet the study objectives: Occupational stress scale, Self-care scale, and psychological flow scale. There validity and reliability were checked.

**Results:**

The study results indicate that self-care and psychological flow levels were low with means of 2.32 and 2.13, while the occupational stress level was high with a mean of 3.91. Also, there are a predictive power of self-care and psychological flow in occupational stress among nurses.

**Conclusions:**

This study shows that of self-care and psychological flow predict occupational stress among nurses, which helps us to manage the occupational stress among them by giving them self-care and psychological flow practices and healing time during work by group counseling and lectures.

## Introduction

Working in hospitals, especially for nursing staff, requires dealing with various cases and direct or indirect interaction and contact with patients. This may put numerous challenges and difficulties for nurses working in hospitals, leading to negative psychological and health consequences due to occupational stress. Therefore, it is highly important to balancing work and life, and practicing self-care to achieve normal psychological flow. These practices could protect from psychological and professional problems and disorders.

Occupational Stress is considered one of the key factors pressures in work environment that can lead to psychological, behavioral, or physiological manifestations of stress and long-term health effects. It is the physical and emotional responses that occur when work requirements exceed the capabilities, resources, or needs of the worker ( Levy et al., 2018).

Occupational stress could lead to developing more severe psychological disorders such as burnout, depression, emotional exhaustion, and others. Therefore, it is crucial to find methods that enable individuals to continue working, reduce human and material losses, and adapt ( Hricova et al., 2020).

Jachens et al. (2018) identified factors that specifically affect humanitarian work and interact with occupational stress, including emergency culture, rewards (e.g. feelings of achievement and pride, constant and unpredictable changes in work, high job involvement, and the inability to withdraw when needed unrealistic job demands) limited availability of social support networks, and insufficient self-care practices.

Self-care practices include activities that help people participate in to maintain, improve, or restore their health, it’s not only for necessities, but helps create a balance among daily activities, pressures, relaxation, and rest, thus enhancing and preserving human life and promoting well-being ( Khademian et al., 2020). These practices are closely linked to professions that involve caregiving, such as nursing. This is due to the nature of the work, which requires engagement in environments where they are continually exposed to emergencies and the distressing stories and experiences of the patients they serve. Along with accompanying feelings such as anger, fear, despair, helplessness, and powerlessness that expose them to high levels of occupational stress ( Santana et al., 2020; Li et al., 2021).

The self-care concept involves taking care of oneself, striving for personal well-being, and making healthy decisions that contribute to happiness by addressing emotional, social, spiritual, and professional aspects; which play a significant role in achieving psychological flow by high level of focus and full engagement in life's activities, enjoying them, and feeling challenged. This demonstrates a correlational relationship between self-care and psychological flow, whereby achieving and maintaining self-care enhances the opportunity to reach a state of psychological flow ( Al-Hiary, 2023).

Psychological flow is defined as a deep state of immersion in enjoyable activities, where individuals perceive their performance as enjoyable and successful, viewing the activity as worthwhile even if it does not lead to further achievement ( Csikszentmihalyi, 2014). Psychological flow is an internal psychological state that engulfs the individual in the activity he/she is engaged in, with a sense of accomplishment in dealing with these activities. From this perspective, flow is an aggregate of psychological factors including focus, control, awareness, motivation, self-awareness, clarity of goals, clear feedback, balance, and challenge. Flow is vital in the workplace, where individuals who frequently experience flow tend to have high job satisfaction, more productivity, and less occupational stress ( Martínez-Zaragoza et al., 2017).

### Research problem

The nursing workforce plays a crucial role in healthcare systems worldwide and contributes to community well-being. Working in the nursing sector is complex, hazardous, and sometimes unsafe. Those responsible for the nursing sector should create suitable working conditions for nurses and consider their mental health and well-being since the quality of care provided partially depends on the quality of their work environment ( International Labor Organization and World Health Organization, 2017). Working in nursing may lead to occupational stress and neglect of self-care practices, affecting their psychological flow. Research has indicated that 43% of doctors and nurses in Jordan suffer from high levels of burnout ( Jordanian Ministry of Health, 2020). Additionally, a study by Dehdashti et al. (2018) found that 70% of nurses experience stress.

Nurses experience numerous psychological, social, emotional, and behavioral shocks and challenges in their work, which can impact their psychological flow during work-related tasks ( Ekman & Halpern, 2015).

Furthermore, the importance of self-care for nursing professionals has emerged as a fundamental and necessary component in effectively addressing occupational stress. By providing efficient services to promote mental health, prevent occupational stress, achieve optimal healthcare quality and flow, and develop self-care mechanisms ( Zhang et al., 2021).

Practicing self-care strategies supports mental health and well-being ( Mills et al., 2018). If nurses neglect self-care, it may lead to health problems that can affect their professional and personal life, which sometimes might results in occupational stress and resignation from the nursing profession ( Kelbach, 2021).

Thus, understanding how self-care and flow experience affect individuals can offer insights on what interventions could be used to improve individuals' quality of life, and consequently impacting their professional lives and work quality, thereby contributing to providing comprehensive care services and reducing the risk of occupational stress among nurses ( Burke et al., 2016). This study aims to investigate the predictive power of self-care and psychological flow on occupational stress among nurses in Jordan.

### Research questions

1. What are the self-care, psychological flow, and occupational stress levels among nurses in Jordan?
2. Is there a predictive power of self-care and psychological flow in occupational stress among nurses in Jordan?

## Methods

### Study design

This study follows the quantitative technique by prediction method. A sample of 476 nurses in Amman was collected voluntarily; the informed consent was taken verbally and written. Also, the researchers took approval from the institutions.

### Participants

The volunteering sample consisting of 476 nurses in Amman Jordan was chosen, which is 2.6% of the population. According to Thompson (2012) it is a good sample size in an error level of (0.05). Their age ranged between 26-58 (M=35.6 ±9.14). 189 of them was males while 287 was females.

### Data collection tools

*Occupational stress scale*

The researchers adapted a scale of 21 items from ( Kushal et al., 2018). Participants rated each item on a five-Likert scale, from 1 (I agree to a very small extent) to 5 (I agree to a very big extent). Higher scores reflect a higher level of occupational stress. The researchers extracted the validity and reliability and found that the discriminate evidence ranged between 0.343 and 0.874. Cronbach's alpha was 0.885 ( Shawaqfeh & Almahaireh, 2024).

*Self-care scale*

The researchers adapted a scale of 22 items divided into 4 dimensions (physical, psychological, spiritual, and occupational) from ( Lichner et al., 2018; Jallad, 2021). Participants rated each item on a five-Likert scale, from 1 (strongly disagree) to 5 (strongly agree). Higher scores reflect a higher level of self-care. The researchers extracted the validity and reliability and found that the discriminate evidence ranged between 0.771 and 0.942. Cronbach's alpha ranged between 0.742 and 0.913 ( Shawaqfeh & Almahaireh, 2024).

*Psychological flow scale*

The researchers adapted a scale of 19 items from ( Alakili, 2015; Al-Hiary, 2023). Participants rated each item on a five-Likert scale, from 1 (strongly disagree) to 5 (strongly agree). Higher scores reflect a higher level of psychological flow. The researchers extracted the validity and reliability and found that the discrimination evidence ranged between 0.458 and 0.874. Cronbach's alpha was 0.818 ( Shawaqfeh & Almahaireh, 2024).

### Data collection

The researchers obtained approval for this study from the Institutional Review Board at the University of Jordan. The registration number is 1546/2024/21. The date was 12/5/2024. Anonymized data was collected by online instruments using Google Forms. The data collected between 13-18/5/2024.

### Data analysis

The researchers entered the data into the SPSS data sheet and used IBM SPSS Statistics for Windows, version 22 (IBM Corp., Armonk, N.Y., USA) to analyze the data. Means, standard deviations, Pearson’s correlation, and multi-regression were calculated. There was no missing data because all questions were required to be answered in the Google form.

### Ethics and consent

The researchers obtained verbal and written approval for this study from the Institutional Review Board at the University of Jordan. The registration number is 1546/2024/21. The date was 12/5/2024.

The informed consent was taken verbally and written. Also, the researchers took approval from the institutions.

## Findings and discussion

### Preliminary analyses

Means, standard deviations, and Person correlations between occupational stress, self-care and psychological flow are shown in Table 1.

**Table 1.** Person correlations between the variables.

Variable	Mean	SD	level	1	2	3	Skewness	Kurtosis
1. OS	3.91	0.41	High	-	-	-	0.435	-0.071
2. SC	2.32	0.46	Low	0.390 *	-	-	0.751	-0.010
3. PF	2.13	0.88	Low	0.178 *	0.160 *	-	0.366	-0.216

OS: Occupational stress. SC: Self-care. PF: Psychological flow.

* Correlation is significant at the 0.01 level (2-tailed).

Table 1 shows a negative correlation between Occupational stress and each Self-care and Psychological flow. Also, there is a positive correlation between Self-care and Psychological flow. Furthermore, the Self-care and Psychological flow levels were low. The Self-care mean was (2.32±0.46), and the Psychological flow mean (2.13 ±0.88) while the Occupational stress level was high mean was (3.91 ±0.41). The results show that the data is normally distributed.

This result can be attributed to work pressures and stress experienced by nurses. According to statistics from the Jordanian Ministry of Health, there are only 37 nurses for every 10,000 citizens ( Jordan News Agency, 2017). To cover this shortage, hospitals allow nurses to work multiple shifts in a single day, leading to long working hours, caring for large numbers of patients, and receiving emergency cases. This diminishes their ability to balance home and work requirements and reduces their capacity for self-care ( Suleiman et al., 2019). Consequently, nurses may experience feelings of boredom, routine, dissatisfaction, negativity, and disengagement from required tasks ( Zaki, 2018), along with an inability to keep up with life demands and a lack of satisfaction due to low-income levels ( Al-Hamdan & Bani Issa, 2022). This leads to a decrease in the level state of psychological flow.

The high level of Occupational stress can be explained by nurses lacking self-care skills and self-attention, as well as insufficient time for relaxation, experiencing flow state, and engaging in various activities due to work pressures. Additionally, the inability to express emotions, feelings of depletion, exhaustion, and fatigue result from a lack of Psychological flow skills and empathy toward patients. Nurses may also struggle to share their sad feelings and bear their emotional burdens ( Zito et al., 2016).

The lack of providing self-care programs and recreational activities in hospitals which help nurses cope with and adapt to Occupational stress, along with insufficient social and psychological support ( Suresh, 2013), contributes to the low level of self-care. Nurses are assigned multiple quantitative and qualitative duties and tasks that exceed their capabilities, leading to role conflict due to the shortage of nursing staff in Jordanian hospitals ( Jordanian Ministry of Health, 2019; World Health Organization, 2020).

One of the reasons for high Occupational stress levels and low levels of psychological flow among nurses is their lack of involvement in decision-making processes at their workplaces, significant disparities between financial income, rewards, and required tasks, as well as limited opportunities for promotion and career advancement with unclear paths, particularly in private hospitals. Additionally, conflicts and lack of harmony with supervisors and managers in nursing practice exacerbate internal dissatisfaction and decrease self-esteem ( Kumar et al., 2011). The work environment in hospitals, in general, is perceived as stressful, and nurses in Jordan express dissatisfaction with the nursing work environment ( Suleiman et al., 2019).

### The multi-regression results

To investigate the predictive power of self-care and psychological flow in occupational stress among nurses in Jordan, a regression test was calculated, and Table 2 shows the results.

**Table 2.** Multi-regression test to investigate the predictive power of self-care and psychological flow in occupational stress.

Variable	R	R ^2^	F	F Sig.	Beta	T	T Sig.
Psychological flow	0.460	0.212	63.461	0.000	-0.379	-10.386	0.000
Self-care					-0.114	5.972	0.000

Table 2 shows the predictive power of self-care and psychological flow in occupational stress among nurses in Jordan was 21.2%, while the correlation was 0.460. Also, The Psychological flow has a more substantial influence on occupational stress, and there is a negative relationship between the variables.

The low practice of self-care and relaxation among nurses, along with diminished psychological flow, bring about feelings of sadness, job dissatisfaction, and dissatisfaction with life. This contributes to creating a negative psychological state, negatively impacting quality of life, professional life, and work quality, leading to increased job stress levels. This explains the inverse relationship between psychological flow and self-care with occupational stress. These findings are consistent with studies by ( Jallad, 2021). Jachens et al. (2018) clarified that working in the humanitarian field, such as nursing, interacts with occupational stress, and one of the reasons for its increase is the lack of self-care practice, relaxation, and weak social support.

Working in nursing is a stressful and traumatic job when exposed to severe medical cases. nurses' sense of enjoyment diminishes, making it difficult for them to perceive their performance as enjoyable and successful, especially when patients die during their shifts. Consequently, psychological flow is unattainable in this negative work environment, thereby increasing their occupational stress.

The explanation for 21.2% of the relationship between flow, self-care, and occupational stress can be attributed to the low level of psychological flow and self-care among nurses. They cannot enhance their physical, psychological, emotional, social, spiritual, and professional well-being due to workload burden, time pressure, lack of social and professional support, limited career advancement, and absence of self-care skills such as relaxation, meditation, spiritual practices, self-awareness, and reactive coping. They also struggle with concentration, integration into work, feelings of fatigue, boredom, loss of commitment and perseverance, task diversity, shift work, inability to communicate with others due to work pressure, and lack of work-life balance, resulting in high levels of occupational stress.

## Conclusion

This study shows that there are predictive power of self-care and psychological flow in occupational stress among nurses, which helps us to manage the occupational stress among them by giving them self-care and psychological flow practices and healing time. addition, there is a high level of occupational stress among nurses and the managers and hospital mostly conduct this issue because it affects their job. However, the self-care and psychological flow among them were low which means they don’t give themselves good attention and it may cause many problems and mental illness. Organizations and Hospitals should provide them with more special assistance, support, and training. Thus, future research should consider social support, coping, and job satisfaction as potentially important variables.

### Limitations and future directions

This study was conducted with a sample of nurses in the Capital Amman, Jordan enrolled in 2024. The study results were interpreted from the participants’ responses on self-care, psychological flow, and occupational stress scales. The researchers recommends further research on occupational stress among nurses to define it and to produce a program to decrease it which can give us better quality work. For the managers at the hospitals, it’s important to train the nurses in selfcare and psychological flow to decrease the occupational stress which can help them to be more effective in their jobs. The universities should give the nurses students classes in selfcare and how to improve their psychological flow.

Future research might also study coping, work environment, job satisfaction, and resilience variables. The creation of counselling and guidance programs to improve self-care and psychological flow among nurses may use technological applications. Thus, the researchers recommended to applied this research on other types of samples such as: Doctors and pharmacists. The future research may include the type of hospitals to see if there are differences between public and private, also in another areas.

## Ethics and consent

The researchers obtained verbal and written approval for this study from the Institutional Review Board at the University of Jordan. The registration number is 1546/2024/21. The date was 12/5/2024.

The informed consent was taken verbally and written after the researchers defined the purpose of the study and that they had free will to accept or reject applying the scales. Also, the researchers took approval from the institutions.

## Data Availability

Figshare. Assessing the predictive power of self-care and psychological flow in occupational stress among nurses (Dataset.sav). https://doi.org/10.6084/m9.figshare.25892764.v4 ( Shawaqfeh, 2024) Data is available under the terms of the CC0. **Repository name:** Figshare Checklist for Assessing the predictive power of self-care and psychological flow in occupational stress among nurses, at https://doi.org/10.6084/m9.figshare.25892764.v4 ( Shawaqfeh, 2024)
