# Supplementary material for: Assessing the Predictive Power of Self-Care and Psychological Flow on Occupational Stress in Nurses
Source: F1000Res. 2025 Feb 28;13:641. Originally published 2024 Jun 17. [Version 2] doi: 10.12688/f1000research.152259.2 (PMC11962362; doi:10.12688/f1000research.152259.2)
Supplement: Peer review history

# Reviewer report: Reviewer response for version 2

**DOI:** 10.5256/f1000research.176513.r371452

**Published:** 2025-04-01

**Authors:** Anas khalifeh

**Affiliations:** 1 Community of Mental Health Nursing, Faculty of Nursing-Zarqa University, Az-Zarqa, Zarqa Governorate, Jordan

It is my pleasure to review the manuscript entitled "Assessing the Predictive Power of Self-Care and Psychological Flow on Occupational Stress in Nurses." In this article, the authors conducted a study among nurses in Amman, Jordan, to explore the predictive relationship of self-care and psychological flow with occupational stress. The study highlights a relevant and timely issue in nursing practice, as occupational stress significantly affects nurses’ well-being, job performance, and the overall quality of healthcare delivery. Investigating psychological factors such as self-care and flow contributes to the understanding of mental health challenges in the nursing workforce and offers potential avenues for intervention and support in clinical settings.

 I have a few questions and comments for the authors' consideration.

 General comment: Need to follow author guidelines for Journal in write abstract and the whole paper.

 My comments are as follows:

1. Title: The revised title is concise and reflects the research aim. It clearly communicates that the study investigates the predictive power of self-care and psychological flow on occupational stress. The change from "predictive ability" to "predictive power" improves clarity and aligns with statistical terminology. However, a more specific version such as “Predictive Roles of Self-Care and Psychological Flow in Occupational Stress Among Nurses in Jordan” could enhance clarity. The title does not reflect the geographic context (Jordan), which may be important for readers seeking region-specific data. Including the population and setting would strengthen specificity.
2. Abstract: While it includes research background, purpose, methodology, and conclusions, the abstract lacks statistical details, instrument characteristics, and specific recommendations. It is also somewhat vague and wordy, which weakens clarity. It also contains grammatical errors and some redundancy. The conclusion in the abstract is general and lacks concrete recommendations for practice.
3. The introduction addresses a significant issue—occupational stress among nurses in Jordan. It includes relevant local and global statistics and highlights the relevance of self-care and psychological flow in addressing stress. However: It is too long and sometimes repetitive. The research gap is not sharply defined. Several references are outdated (>10 years). Some ideas (e.g., burnout, self-care importance) are repeated.

The theoretical framework is implicit rather than explicitly stated, although flow theory (Csikszentmihalyi, 2014) is mentioned.

 Research Problem and Purpose: The problem is identified but not succinctly stated. The significance of the study is highlighted through statistics and citations.

 Research Questions: Clearly listed.

 4- Methods

 Design: Quantitative, predictive correlational study using a sample of 476 nurses from Amman. The cross-sectional design is appropriate for prediction but limits causal inference. However, no justification is given for selecting this design over other approaches (e.g., structural equation modeling might have offered deeper insights).

 Setting : No description of hospital type (e.g., public vs. private), work units (e.g., ICU, ER), or shift types (rotational, night), which are major determinants of occupational stress. The reasons for selection.

 Sample: Inclusion/exclusion criteria are not clearly described. Sampling was voluntary and conducted online via Google Forms, potentially excluding older or less tech-savvy nurses, introducing sampling bias.

 The recruitment process is vague—no details on how participants were approached or incentivized.

 Study instrument: Instruments were adapted, not developed, yet authors ambiguously claim both. Cultural validation of tools is not discussed—do the constructs of self-care or psychological flow translate equivalently into Arabic/Jordanian contexts?. Is instrument used in nursing students and valid for this population? Which language they were? Did you get permission to use the instrument?

 Ethical considerations: How do you protect the identity of participants? Need to mention process of data saving. Did you get permission to use the instrument? IRB approval obtained and informed consent described. This was a strong component. However, verbal consent in online surveys raises concerns. How was this recorded? What mechanisms ensured participants understood their rights? No discussion of scale structure (e.g., subscales, factor structure) limits interpretability.

 Data collection procedure: I suggest explaining data collection steps.

1. Results: No tables or narrative summaries of key demographic features (years of experience, ward type, education, income) are presented, which severely limits result interpretation. No demographic characteristics of participants are presented in the results—major omission. Examine gender-based differences (the sample was 60% female)

Gender, age, and experience might moderate stress levels. Discussion does not explore why psychological flow is a stronger predictor. Nor does it explore interaction effects or control for confounding variables. Limitations are acknowledged vaguely and are not integrated into the interpretation of results. Authors do not engage with potential alternative explanations (e.g., work-life balance, leadership support). No attempt to test for mediation or moderation (e.g., Does self-care mediate the relationship between workload and stress?).

 5- Discussion:  much more discussion of findings is needed. Wider background reading is required to set the scene and also generalizability to the wider nursing students. There are findings discussed but not present or highlight in result. Justify the reasons may relate to finding and comparing with other studies. A valuable and interesting piece of research that has the opportunity to be expanded to other nations and the discussion points to direct further areas of research. The discussion would benefit from some additional reorganizing. No concrete or evidence-based interventions are proposed (e.g., mindfulness, organizational changes).

 Implications for “nursing education” and “administration” are mentioned but not elaborated.

 No link back to theoretical framework—missed opportunity to explain why self-care or flow is protective from a psychological model.

 The authors missed discussing institutional and policy-level changes, which is important given systemic stressors in Jordanian hospitals.

 6- Recommendations:

 Are broad and lack actionability.

 “Train nurses in flow” – How? Through workshops, CBT, mindfulness?

 “Conduct programs” – What type of programs? What content? What frequency?

 7- Conclusion: Add critical findings and conclusion.

 8- Implications: Add implications. No structured plan for implementation or cost-benefit analysis is provided.

 9- Limitation: Briefly acknowledged, but many are missing, including:

 Lack of subgroup analysis (e.g., by ward type or shift)

 Non-response bias

 Use of only self-report tools

 Theoretical and cultural limitations

Is the work clearly and accurately presented and does it cite the current literature?

Partly

If applicable, is the statistical analysis and its interpretation appropriate?

Partly

Are all the source data underlying the results available to ensure full reproducibility?

Partly

Is the study design appropriate and is the work technically sound?

Yes

Are the conclusions drawn adequately supported by the results?

Partly

Are sufficient details of methods and analysis provided to allow replication by others?

No

Reviewer Expertise:

nursing

I confirm that I have read this submission and believe that I have an appropriate level of expertise to confirm that it is of an acceptable scientific standard, however I have significant reservations, as outlined above.

# Reviewer report: Reviewer response for version 1

**DOI:** 10.5256/f1000research.166997.r349181

**Published:** 2024-12-26

**Authors:** Ahmad Al-Sagarat

**Affiliations:** 1 Community and Psychiatric Nursing Department, Faculty of Nursing, Mutah University, AL-Karak, Jordan

The article is searching one of the most issues that are facing the Jordanian nurses specifically. In some areas I would like the author to use the most recent studies and avoid using old literature older than 10 years old. I would like the author to expand more on the limitations from different perspectives such as: sample, type of hospital, different geographic areas, mixed method--- etc. Moreover, I would like the author to add the implication of this study for nursing practice, nursing administration, nursing education and future research. Finally, the overall article is highly recommended and approved  for indexing.

Is the work clearly and accurately presented and does it cite the current literature?

Partly

If applicable, is the statistical analysis and its interpretation appropriate?

Yes

Are all the source data underlying the results available to ensure full reproducibility?

Yes

Is the study design appropriate and is the work technically sound?

Yes

Are the conclusions drawn adequately supported by the results?

Yes

Are sufficient details of methods and analysis provided to allow replication by others?

Yes

Reviewer Expertise:

Mental health nursing

I confirm that I have read this submission and believe that I have an appropriate level of expertise to confirm that it is of an acceptable scientific standard.

# Response

**Published:** 2024-12-30

**Authors:** Abdallah Almahaireh

**Affiliations:** The University of Jordan, Amman, Amman Governorate, Jordan

Dear Ahmad Al-Sagarat

 We wanna thank you for these notes that improve our work.

 We made the changes you reported and used recent references as we could.

 Best Regards,

# Reviewer report: Reviewer response for version 1

**DOI:** 10.5256/f1000research.166997.r301709

**Published:** 2024-07-23

**Authors:** Amal I Khalil

**Affiliations:** 1 psychiatric nursing, king Saud bin Abdelaziz university for health sciences, college of nursing, Jeddah, Makkah Province, Makkah Province, Saudi Arabia; 2 College of Nursing, King Saud Bin Abdul-Aziz University for Health Sciences, Riyadh, Saudi Arabia

thank you for giving the opportunity to review this manuscript let's start with the title: :

 despite it is concise but it is little bit unclear for the reader so i suggest to be changed into  

- "Predictive Roles of Self-Care and Psychological Flow in Occupational Stress Among Nurses" or 
- "Assessing the Predictive Power of Self-Care and Psychological Flow on Occupational Stress in Nurses"

Abstract :As a peer reviewer, here are some potential drawbacks and areas for improvement in the abstract:

1.  The abstract is slightly wordy and can be more concise. This can be achieved by removing redundant phrases and ensuring each sentence adds new information.
2.  The objective of the study could be more clearly stated at the beginning of the abstract. Currently, it is embedded within a broader discussion of the context.
3.  The abstract mentions that three scales were developed, but it does not provide any details on their development, validation, or reliability. Including brief information on these aspects would strengthen the abstract.
4. The results section mentions that self-care and psychological flow levels were low, and occupational stress was high, but it does not provide any specific data or statistics to support these claims. Including key statistics would make the results more robust.
5. : The conclusion mentions that self-care and psychological flow practices can help manage occupational stress but does not provide specific recommendations or implications for practice. Adding this information would make the conclusions more actionable.
6.  The abstract contains some grammatical insufficiency and awkward phrasing that can be refined for clarity and readability.
7. **Introduction **:Here are some potential drawbacks of the provided introduction and suggestions for improvement:
8.  The introduction is quite lengthy and contains many ideas that could be more concisely presented. A more focused introduction would help readers quickly grasp the main points.
9. The introduction would benefit from clearer structure and organization. It jumps between different concepts without clear transitions. Organizing it into distinct paragraphs for context, problem statement, and the purpose of the study would improve readability.
10. Some points are repeated, such as the importance of self-care and psychological flow, which can be consolidated to avoid redundancy.
11.  While the introduction provides a lot of background information, it sometimes strays from the main focus of the study. Keeping the information relevant to the research question would make the introduction more impactful.
12.  The introduction includes many references, which, while supporting the points made, can overwhelm the reader. Select the most pertinent references to strengthen the key points.
13. The introduction could benefit from more specific statements about the research gap and the need for this study. It should more clearly highlight what is unknown or understudied about the relationship between self-care, psychological flow, and occupational stress among nurses.
14. **research problem  it is better to say significance of the study and here some drawbacks:**
15. **Length and Conciseness**: The problem statement is quite long and includes a lot of background information that could be more concisely presented. The main problem could be stated more directly.
16. **Clarity and Focus**: The statement introduces several related issues (e.g., burnout, emotional exhaustion, psychological pressure, self-care, psychological flow) without clearly connecting them to the main research problem. Focusing on the central issue of how self-care and psychological flow impact occupational stress among nurses would improve clarity.
17. **Redundancy**: Some points are repeated, such as the impact of occupational stress and the importance of self-care. These could be combined to streamline the text.
18. **Lack of Specificity**: The problem statement mentions various statistics and studies but does not clearly state the specific gap or need that the current study addresses. More emphasis on what is unknown or understudied would be beneficial.
19. **Transition and Structure**: The transitions between different points could be smoother. Organizing the statement into distinct paragraphs for background, problem identification, and the purpose of the study would enhance readability.
20. There are many references to other studies, which, while supporting the points made, can make the problem statement
21. **Methods: first the definition of variables  "self care , psychological flow, occupational stress is important  for the readers ,the other thing Sample Representativeness**: The sample, while sizable, represents only 2.6% of the nurse population in Amman. This could limit the generalizability of the findings to all nurses in Jordan, and how they calculate the sample size??The study's cross-sectional design limits the ability to draw causal inferences. While predictive relationships can be identified, causal conclusions cannot be definitively made. 
22. the scales used were adapted from other studies which may differ culturally as well the authors mention developed which is wrong as they adapted both scales
23. **Data Collection Method**:
  - Using online instruments such as Google Forms may exclude participants who are less tech-savvy or have limited internet access, potentially introducing sampling bias.
24. **Limited Contextual Information**:
  - The study might benefit from including more contextual information about the work environment, workload, and other stressors specific to the nursing profession in Jordan. These factors could provide a deeper understanding of occupational stress.
25. **Ethical Considerations**:
  - While informed consent was obtained verbally and in writing, ensuring that participants fully understand the study's aims and their rights is crucial. Detailed information on how consent was obtained and documented would enhance ethical transparency.he study does not account for other variables that might influence occupational stress, such as personal life stressors, job satisfaction, or organizational support. Including these factors could provide a more comprehensive analysis. as the authors didn't include demographic characteristics of the participants which is very important in interpreting the results 
  - also short data collection time   (13-18/5/2024) might not capture variations in stress levels that could occur over different times or under different circumstances.
  - results of the study: missed Demographic characteristics of the participants it is better to separate results from discussion for more interpretations and discussions 
  - big number of cited references are old and needs updates
  - where is the recommendations and clinical implications of these results 
  - a lot of limitations should be recognized in this study such as : self reported tools, lack of generalizability since the study was conducted only on Jordanians living in Amman, mediating variables should be considered as mentioned before

Is the work clearly and accurately presented and does it cite the current literature?

Partly

If applicable, is the statistical analysis and its interpretation appropriate?

Partly

Are all the source data underlying the results available to ensure full reproducibility?

Yes

Is the study design appropriate and is the work technically sound?

Partly

Are the conclusions drawn adequately supported by the results?

Yes

Are sufficient details of methods and analysis provided to allow replication by others?

Yes

Reviewer Expertise:

psychiatric, psychological and psychosocial research all types of research designs as well child ,adult ,and elderly psychological and psychiatric disorders.

I confirm that I have read this submission and believe that I have an appropriate level of expertise to confirm that it is of an acceptable scientific standard, however I have significant reservations, as outlined above.

# Response

**Published:** 2024-12-30

**Authors:** Abdallah Almahaireh

**Affiliations:** The University of Jordan, Amman, Amman Governorate, Jordan

Dear Amal I Khalil,

 We wanna thank you for these notes that improve our work.

 We made the changes you reported.

 and Added a reference for the sample size.

 Best Regards,
